# Inverse regulation of two classic Hippo pathway target genes in *Drosophila* by the dimerization hub protein Ctp

**DOI:** 10.1038/srep22726

**Published:** 2016-03-14

**Authors:** Daniel A. Barron, Kenneth Moberg

**Affiliations:** 1 Department of Cell Biology, Graduate Program in Biochemistry, Cell and Developmental Biology, Emory University School of Medicine, Atlanta, GA 30322, USA; 2 Department of Cell Biology, Medical Scientist MD/PhD Training Program, Emory University School of Medicine, Atlanta, GA 30322, USA

## Abstract

The LC8 family of small ~8 kD proteins are highly conserved and interact with multiple protein partners in eukaryotic cells. LC8-binding modulates target protein activity, often through induced dimerization via LC8:LC8 homodimers. Although many LC8-interactors have roles in signaling cascades, LC8’s role in developing epithelia is poorly understood. Using the *Drosophila* wing as a developmental model, we find that the LC8 family member Cut up (Ctp) is primarily required to promote epithelial growth, which correlates with effects on the pro-growth factor dMyc and two genes, *diap1* and *bantam*, that are classic targets of the Hippo pathway coactivator Yorkie. Genetic tests confirm that Ctp supports Yorkie-driven tissue overgrowth and indicate that Ctp acts through Yorkie to control *bantam* (*ban*) and *diap1* transcription. Quite unexpectedly however, Ctp loss has inverse effects on *ban* and *diap1*: it elevates *ban* expression but reduces *diap1* expression. In both cases these transcriptional changes map to small segments of these promoters that recruit Yorkie. Although LC8 complexes with Yap1, a Yorkie homolog, in human cells, an orthologous interaction was not detected in *Drosophila* cells. Collectively these findings reveal that that *Drosophila* Ctp is a required regulator of Yorkie-target genes *in vivo* and suggest that Ctp may interact with a Hippo pathway protein(s) to exert inverse transcriptional effects on Yorkie-target genes.

The LC8 family of cytoplasmic dynein light-chains, which includes vertebrate LC8 (aka DYNLL1/DYNLL2) and *Drosophila* Cut-up (Ctp), are small highly conserved proteins that are ubiquitously expressed and essential for viability^1^,^2^,^3^,^4^. The LC8 protein is 8 kilodaltons (kD) in size and was first identified as an accessory subunit in the dynein motor complex, within which an LC8 homodimer binds to and stabilizes a pair of dynein intermediate chains (DIC)^1^,^5^,^6^. However, the LC8 protein has since emerged as a general interaction hub with multiple dynein/motor-independent roles and binding partners^3^,^7^,^8^. In fact the majority of LC8 protein in mammalian cells is not associated with either dynein or microtubules^1^, and LC8 orthologs are encoded in the genomes of flowering plants that otherwise lack genes encoding heavy-chain dynein motors^9^.

Accumulating evidence has reinforced the idea that the primary role of LC8 in mammalian cells is to facilitate dimerization of its binding partners via LC8 self-association, a mechanism that has been termed ‘molecular velcro’^7^. LC8 can be found in association with over 40 proteins that function in diverse cellular processes, including intracellular transport, nuclear translocation, cell cycle progression, apoptosis, autophagy, and gene expression^8^,^10^. LC8 is found in both the nucleus and cytoplasm and can interact with partners in either compartment. For example the mammalian kinase Pak1 binds and phosphorylates LC8 in the cytoplasm, which in turn enhances the ability of LC8 to interact with the BH3-only protein Bim and inhibit its pro-apoptotic activity^11^,^12^. Accordingly, overexpression of LC8 or the phosphorylation of LC8 by Pak1 enhances survival and proliferation of breast cancer cells in culture^12^. LC8 also binds estrogen receptor-α (ERα) and facilitates ERα nuclear translocation, which in turn recruits LC8 to the chromatin of ERα-target genes^13^,^14^,^15^. In the cytoplasm, LC8 is also found in association with the kidney and brain expressed protein (KIBRA), which is an upstream regulator of the Hippo tumor suppressor pathway^16^. KIBRA binding potentiates the effect of LC8 on nuclear translocation of ERα, suggesting crosstalk may occur between LC8-regulated pathways^15^. The KIBRA-LC8 complex also interacts with Sorting Nexin-4 (Snx4) to promote dynein-driven traffic of cargo between the early and recycling endosomal compartments^17^. Thus, LC8 has been linked to a variety of proteins in both the cytoplasm and nucleus that play important roles in signaling, membrane dynamics, and gene expression.

*Drosophila* Ctp differs from vertebrate LC8/DYNLL by only four conservative amino acid substitutions across its 89 amino acid length. Similar to mammalian LC8, phenotypes produced by Ctp loss in flies imply roles in multiple developmental mechanisms. *Drosophila* completely lacking Ctp die during embryogenesis due to excessive and widespread apoptosis^2^,^18^. Partial loss of Ctp function causes thinned wing bristles and morphogenetic defects in wing development, as well as ovarian disorganization and female sterility^2^. Within salivary gland cells, Ctp promotes autophagy during pupation^19^, while in neuronal stem cells it localizes to centrosomes and influences mitotic spindle orientation and the symmetry of cell division^20^. Testes mutant for *ctp* have motor-dependent defects in spermatagonial divisions as well as motor-independent defects in cyst cell differentiation^21^. A recent study linked *ctp* mRNA expression to the zinc-finger transcription factor dASCIZ and showed that knockdown of either Ctp or dASCIZ reduces wing size^22^. In sum, this diversity of effects produced by Ctp loss in different *Drosophila* cell types suggest that Ctp plays important yet context specific roles *in vivo*. However our knowledge of molecular pathways that require Ctp, and in turn underlie these developmental phenotypes associated with Ctp loss, remain poorly characterized.

Here we use a genomic null allele of *ctp* and a validated *ctp* RNAi transgene to assess the role of the Ctp/LC8/DYNLL protein family in pathways that act within the developing *Drosophila* wing epithelium. We find that clones of *ctp* null cells are quite small relative to controls and that RNAi depletion of Ctp shrinks the size of the corresponding segment of the adult wing without clear defects in mitotic progression or tissue patterning. The effect of Ctp depletion on adult wing size is primarily associated with a reduction in cell size, rather than cell division or cell number, implying a role for Ctp in supporting mechanisms that enable developmental growth. In assessing the effect of Ctp loss on multiple pathways that control wing growth, we detect robust effects on one–the Hippo pathway. The Hippo pathway is a conserved growth suppressor pathway that acts via its core kinase Warts to inhibit nuclear translocation of the coactivator Yorkie (Yki), which otherwise enters the nucleus, complexes with the DNA-binding factor Scalloped (Sd), and activates transcription of growth and survival genes^23^,^24^,^25^,^26^. In parallel to the effect of Ctp loss on clone and wing size, Ctp loss alters expression of the classic Yki target genes *bantam* and *thread(th)/diap-1* in wing pouch cells. Parallel genetic tests confirm a requirement for *ctp* in Yki-driven tissue growth in the wing or eye. Quite unexpectedly however, Ctp loss has opposing effects on *bantam* and *diap1* transcription in wing pouch cells: *bantam* transcription is strongly elevated while *diap1* expression is strongly decreased in cells lacking Ctp. In each case, these effects map to small segments of DNA in the *ban* and *diap1* promoters that recruit Yki transcriptional complexes^24^,^25^,^27^. Epistasis experiments confirm that Yki is required to activate the *bantam* promoter in Ctp-depleted cells, and that transgenic expression of Yki can overcome the block to *diap1* transcription. In sum these data argue that Ctp supports physiologic Hippo signaling in wing disc epithelial cells, and that Ctp likely interacts with an as yet unidentified Hippo pathway protein(s) to exert inverse transcriptional effects on Yorkie-target genes. These types of inverse effects have not previously been described within the Hippo pathway, and imply that distinct subsets of genes within the Yorkie transcriptome can be simultaneously activated and repressed in developing tissues via a mechanism that involves Ctp.

## Materials and Methods

### Fly Strains

All crosses were maintained at 25 °C unless otherwise noted. Alleles used in these studies (Bloomington stock number indicated) are as follows: *thread-lacZ* (#12093), *ex697* (*ex-lacZ*, #44248), *e2f1rM729* (*e2f1-lacZ*, #34054), *UAS-yki-V5* (#28819), yki^B5^ (#36290), *mirr*^*DE*^*-Gal4* (#29650), *EcRE-lacZ* (#4517), *Stat92E-10XGFP* (#26198) (courtesy of R.Read), *UAS-diap1* (#6657), *UAS-p35* (#5073), *UAS-dcr2;enGal4,UAS-GFP* (#25752) obtained from the Bloomington *Drosophila* Stock Center. *UAS-yki-IR* (v104523) and *UAS-ctp-IR* (v43116) were obtained from the Vienna Drosophila Resource Center (VDRC). Other alleles used were *ctp*^*ex3*^ (Gift of Bill Chia), *enGal4/CyO, HRE-lacZ, HREmut6-lacZ, 2B2-lacZ, 2B2C-lacZ* (all gift of D.J. Pan)*, brC12-lacZ* (K. Irvine), *GMR-Gal4, UAS-ykiS168A:GFP* (K. Harvey), *E-spl(m)ß-CD2, Su(H)-lacZ*, *PCNA-GFP*, *ban-3xGFP*, and *UAS-yki* (D.J. Pan).

### Immunofluorescence Microscopy

Immunostaining and confocal microscopy performed as described previously on a Zeiss 510 inverted confocal microscope^28^. Primary antibodies include mouse anti-β-Gal 1:1000 (Promega); rabbit anti-LC8 (1:100) (W. Sale), mouse anti-Ci (1:50), mouse-α-Notch 1:10, mouse anti-Wg (1:800), mouse anti-Cut (1:100) and mouse anti-DIAP1 (1:50) (DSHB); rabbit α-yki 1:1000 (K. Irvine); mouse α-rat CD2 1:100 (Research Diagnostics, Inc.); mouse anti-V5 (1:200, Invitrogen); mouse anti-dpERK (1:10,000, Sigma); rabbit anti-phospho histone H3 (1:100), rabbit anti-phospho-Smad1/5 (1:100), rabbit anti-DCP1 (1:150), rabbit anti-Caspase3 (1:100) (Cell Signaling); mouse anti-dMyc (monoclonal P4C4-b10); mouse anti-Cyclin E (H. Richardson). BrdU assays performed as described previously (Robinson *et al*. 2010) with mouse anti-BrdU (1:100; Becton Dickinson).

### Wing/Eye Measurements

Eyes/wings were imaged on a Leica DFC500 CCD camera and quantified with Image J. Posterior compartment ratio (PCR) = posterior compartment size/total wing size.

## Results

### Ctp is required for imaginal-disc derived adult tissues to grow to normal size

**Figure 1:**
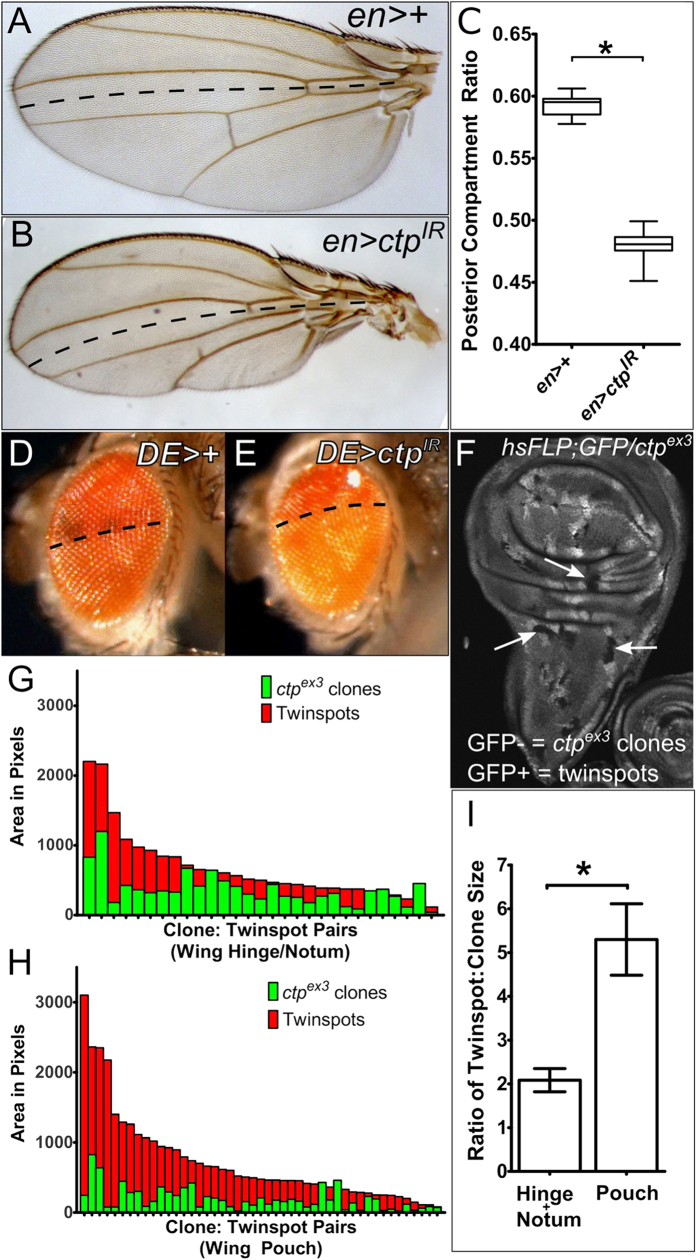
c*tp* loss reduces compartment size and clonal growth. Paired images of control (**A,D**) and Ctp-depleted (**B,E**) adult wings and eyes generated using the *en-Gal4* (**A,B**) or *mirr*^*DE*^*-Gal4* (*AKA dorsal eye (DE)-Gal4*) (**D,E**) drivers in combination with the *ctp*^*IR*^ transgene. *en* is expressed in posterior wing cells (below dotted line in (**A,B**)). *DE* is expressed in dorsal eye cells (above dotted line in (**D,E**)). (**C**) Box-plot quantitation of the wing posterior compartment ratio (PCR = P area/P + A areas) of the genotypes in (**A**) (*n* = 22) and (**B**) (*n* = 31). Standard error of the mean (SEM) is indicated. *p < *0.0001*. (**F**) Confocal image of a *ctp* mosaic L3 wing disc (*ctp*^*ex3*^*,FRT19A/ubi-GFP,FRT19A,hsFlp*) containing GFP-negative *ctp*^*ex3*^ clones (arrows) and their paired GFP-positive twinspots. (**G,H**) Histogram plots of the two-dimensional area of multiple *ctp*^*ex3*^ clone:twinspot pairs in the hinge/notum (**G**) (*n* = 29) or pouch (**H**) (*n* = 47) of L3 wing discs. Each pair of bars on the X-axis represents a distinct clone:twinspot pair. The clones are significantly smaller than their paired twinspots, *p *=* *0.0002* for (**G**), *p < *0.0001* for (**H**). (**I**) Bar graph plot of average ratios of *ctp*^*ex3*^ clone:twinspot sizes in the hinge/notum versus the pouch. SEMs are indicated. *p *=* *0.0034*.

To test the role of *ctp* in the developing wing disc, a *UAS* transgene encoding a *ctp* RNA interference (RNAi) cassette (Vienna line #43116) was expressed in the posterior compartment of the larval wing disc at 25 °C using the *engrailed-Gal4* driver (*enGal4*). Depletion of Ctp protein in posterior cells of *en* > *ctp-IR* discs was confirmed by immunostaining with an antibody raised against the *Chlamydomonas reinhardtii* homolog of Ctp that also cross-reacts with metazoan LC8/Ctp proteins^1^ (Figure S1). Ctp-depleted larval wing discs develop into adult wings with a fully penetrant phenotype of a shrunken posterior blade (Fig. 1A–C). This effect occurs without obvious scarring of the adult wing or major disruption in its pattern of venation, with the exception of occasional truncated cross veins (e.g. wing in Fig. 1B). Ctp-depletion with a second larval driver, *mirr*^*DE*^*-Gal4*, which is active in the dorsal half of developing larval discs^29^, shrinks the dorsal half of the adult eye (more darkly pigmented region above the dotted lines in Fig. 1D,E), contracts the dorsal surface of the adult head, and leads to small, thin thoracic bristles (Figure S2). The Ctp-RNAi small-bristle phenotype matches a thin-bristle phenotype observed in adult flies carrying the hypomorphic *ctp* mutation, *ctp*^*ins1*^, and further confirms the validity of the RNAi cassette^2^. As in the wing, Ctp depletion did not obviously scar the dorsal domain of the adult eye or strongly disrupt its surface organization, suggesting that Ctp depletion impairs growth of these organs but less so the mechanisms that drive their morphological patterning.

To better assess the effects of Ctp loss on cell proliferation and survival, *ctp* mutant clones (GFP-) and their wildtype twinspots (GFP+) were generated by combining a heatshock(hs)-induced Flp mitotic recombination system with the *ctp*^*ex3*^ null allele^2^. Within dissected larval wing discs, GFP-deficient *ctp*^*ex3*^ clones could be found in the pouch, hinge and notum at 48 hrs post-clone induction (Fig. 1F). In the hinge and notum, these *ctp* null clones can reach a fairly large sizes (see arrows in Fig. 1F) but are nonetheless ~2-fold smaller than their control twinspots (Fig. 1F,G,I). This trend toward small clones is more pronounced in the pouch where *ctp*-null clones are occasionally missing and consistently quite small relative to their age-matched twinspots (e.g. see Fig. 1F). A quantitative measurement across multiple pouch clone pairs with extant twinspots shows an approximate 5-fold reduction in *ctp* mutant clone area (Fig. 1H,I). This apparently unequal growth deficit elicited by *ctp* loss in hinge/notum cells vs. pouch cells could nonetheless stem from perturbation of an otherwise ubiquitous cellular process, such as the mitotic spindle defects observed in *ctp* mutant neuronal stem cells^20^. Alternatively, it could indicate a role for Ctp in a molecular pathway or process that is especially important in pouch cells, similar to the cell-type specific and motor-independent requirement for *ctp* in germline and somatic cyst cells^21^.

### Ctp loss elevates apoptosis and division of wing disc cells and reduces their size

**Figure 2:**
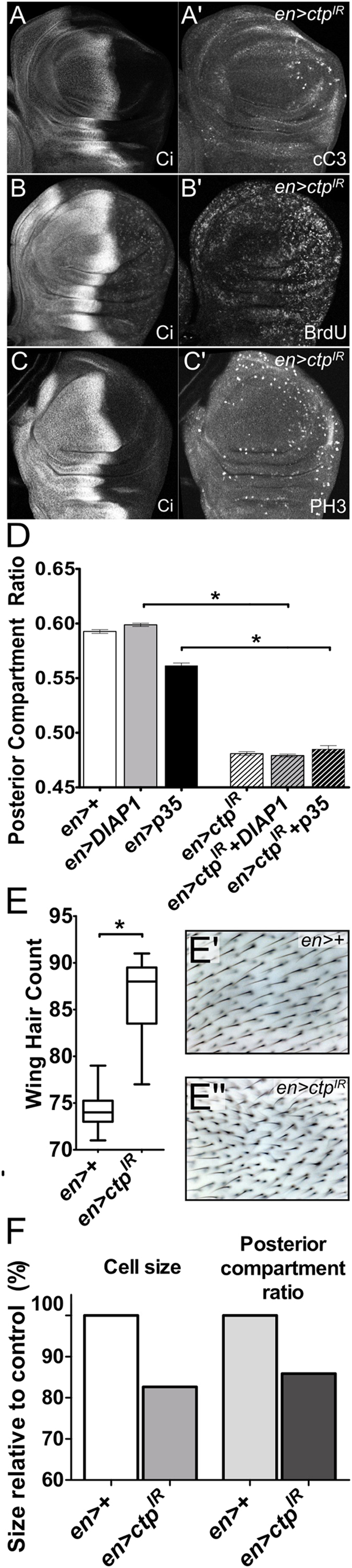
Effects of Ctp loss on the division, survival, and size of wing cells. Confocal images of *en* > *ctp*^*IR*^ L3 wing discs stained for (**A′**) cleaved Caspase-3 (cC3), (**B′**) the base analog BrdU, or (**C′**) phospho-histone H3 (PH3). Anti-Cubitis interruptus (Ci) staining marks the anterior domain (**A–C**). (**D**) Quantitative analysis of PCR in the indicated genotypes. *en* > *diap1* (*n* = 13) is significantly larger than *en* > *diap1* + *ctp*^*IR*^ (*n* = 12) (*p < *0.0001*), and *en* > *p35* (*n* = 10) is significantly larger than *en* > *p35* + *ctp*^*IR*^ (*n* = 17) (*p < *0.0001*). (**E–E″**) Box-plot quantitation and images of wing hair cell density in a fixed area in the posterior wing between the L4 and L5 wing veins in control (*en* > + ) (*n* = 10) or Ctp-depleted (*en* > *ctp*^*IR*^) (*n* = 9) adult wings. SEMs are indicated. *p < *0.0001*. (**F**) Comparative effects of Ctp-depletion (*en* > + vs. *en* > *ctp*^*IR*^) on cell size (data in 2E) and PCR (data in 1C). *en* > + values are standardized to 100%.

To better assess the effects *ctp* loss on division and survival of larval wing disc cells, larval wing cells depleted of Ctp were probed with antibodies to cleaved caspase-3 (cC3), the incorporated nucleotide base analog bromodeoxyuridine (BrdU), and the mitotic marker phospho-histone H3 (pH3) (Fig. 2A–C). cC3 is moderately elevated in the posterior compartment of *en* > *ctp-IR* wing discs (Fig. 2A,A′), and an antibody to the cleaved caspase Dcp-1 detects elevated apoptosis in *ctp*^*ex3*^ mutant wing pouch cells (Figure S3). This finding in disc cells parallels the widespread apoptosis that occurs in *ctp* mutant embryos^2^ and raises the possibility that death of *ctp* mutant cells may deprive the developing wing of a sufficient pool of cells to achieve normal size. To test this hypothesis, two transgenes encoding either the *Drosophila* protein Diap1, which blocks caspase cleavage, or the baculoviral protein p35, which inhibits active cleaved caspases^30^, were co-expressed with *ctp-IR* from the *en* driver (Fig. 2D). Neither of these anti-apoptotic transgenes were able to significantly rescue small *en* > *ctp-IR* wings, indicating that cell death is unlikely to be the sole cause of wing blade undergrowth induced by chronic Ctp-depletion.

The early mitotic marker phospho-histone H3 (pH3) is increased among Ctp-depleted larval wing pouch cells (Fig. 2C,C′). Taken in isolation, this higher abundance of pH3-positive *ctp-IR* larval wing cells could be indicative of an accelerated rate of mitotic entry or a slower rate of M-phase transit. Although disrupting LC8 function in cultured mammalian cells does not impede mitotic progression^31^, a past study in *Drosophila* attributed an excess-pH3 phenotype in Ctp-depleted cells to dynein-motor defects that block cells in mitosis and thus enrich for M-phase markers^22^. This mitotic-block model logically predicts that Ctp-depleted cells accumulate in M-phase and thus depopulate other phases of the cell cycle. However, BrdU-incorporation analysis of *ctp-IR* wing pouch cells does not show a depletion of S-phase cells and in fact seems to show an increase in the frequency of S-phase entry (Fig. 2B,B′). A very similar increase in cell division occurs in *Drosophila* germ cells lacking *ctp*^21^. Intriguingly *ctp* loss reduces cell division among neural stem cells^32^, suggesting that Ctp plays distinct proliferative roles in different cell types. In combination, the pH3 and BrdU data presented here argue that Ctp-depleted larval wing disc cells are able to actively transit between the mitotic and DNA synthesis phases of the cell cycle, suggesting that a cell cycle block is not the prime cause of small *ctp-IR* adult wings.

Genetic manipulations that reduce cell size can also shrink *Drosophila* adult organs. Thus, the lack of experimental evidence pointing to excess apoptosis or a proliferative deficit among Ctp-depleted cells prompted analysis of the effect of Ctp loss on cell size. Each cell in the adult wing generates a single hair, enabling a quantitative determination of cell density derived from hair counts within a fixed region of the adult wing. Applying this approach to an area of the posterior wing blade between the L4 and L5 veins reveals that *en* > *ctp-IR* wings have significantly higher hair density relative to control *en* > + wings, indicating that Ctp-depleted cells are smaller (Fig. 2E). The measured effect of *en* > *ctp-IR* depletion on cell size (~14% smaller; Fig. 2E) is of similar magnitude to its effect on the relative size of the posterior wing blade (~17% smaller; Fig. 1C), indicating that effects on cell size, rather than cell number, are likely be a central cause of small *en* > *ctp-IR* wings (Fig. 2F).

**Figure 3:**
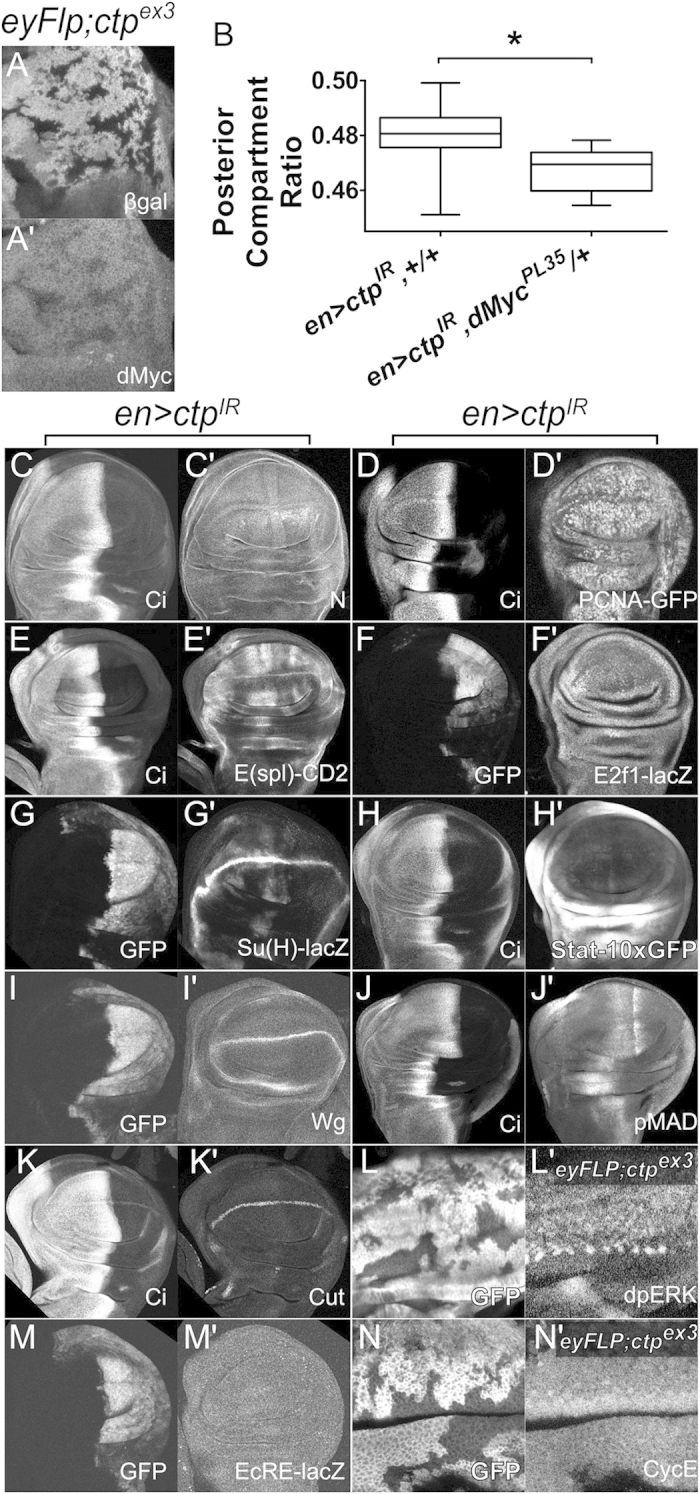
Effect of Ctp loss on a panel of disc cell proliferation and/or growth pathways. (**A**) Confocal image of an L3 eye disc (posterior to the right) mosaic for *ctp*^*ex3*^ clones marked by the absence of β-galactosidase (βgal) (**A**) and stained for dMyc (**A′**). Note the drop in anti-dMyc fluorescence in *ctp*^*ex3*^ clones in (**A′**). (**B**) Quantitation of PCR in the indicated adult genotypes shows that heterozygosity for the *dMyc*^*PL35*^ allele (*n* = 6) has a dominant enhancing effect on the *en* > *ctp*^*IR*^ phenotype (*n* = 31). SEMs are indicated. *p *=* *0.0037*. (**C–K, M**) Confocal images of L3 wing discs with Ctp depleted in the posterior domain (right-side in all images, with Ci marking the anterior domain (**C–E,H,J,K**) or transgenic *UAS-GFP* marking the posterior domain (**F,G,I,M**)) and analyzed for each of the indicated factors: (**C′**) anti-Notch (N) to detect the C-terminal fragment of the Notch receptor; (**D′**) the E2F-activity reporter *PCNA-GFP*; (**E′**) the Notch-activity reporter *E(spl)mβ-CD2*; (**F′**) the *E2f1-lacZ* enhancer trap; (**G′**) the Notch-activity reporter *Su(H)-lacZ*; (**H′**) the Jak-Stat activity reporter *Stat10x-GFP*; (**I′**) anti-Wingless (Wg) to detect the ligand of the Wg/Wnt pathway; (**J′**) anti-phospho-Mothers against decapentaplegic (Mad) to detect signaling through the Dpp pathway; (**K′**) anti-Cut, a target of both Wg and Notch; (**M′**) the ecdysone receptor (EcR) reporter activity *EcRE-lacZ*. (**L,N**) L3 eye discs mosaic for *ctp*^*ex3*^ clones marked by the absence of GFP (**L,N**): (**L′)** anti-diphospho-Erk to detect signaling downstream of the *Drosophila* epidermal growth factor receptor (DER); (**N′**) anti-CyclinE (CycE), which is rate-limiting for progression into S-phase.

The smaller cell size of Ctp-depleted wing cells combined with the shorter, thinner thoracic bristles seen with *mirr*^*DE*^*-Gal4* driven Ctp (Figure S2) is reminiscent of phenotypes induced by mutations in genes that support metabolic process of growth, such as the *diminutive* gene or the *Minute* ribosomal RNA genes^33^ (and reviewed in ref. 34). The *diminutive* (*dm*) locus encodes the *Drosophila* Myc protein (dMyc), a well-established pro-growth transcription factor that promotes cell and tissue growth^35^. Notably, *ctp*^*ex3*^ null clones generated in the eye disc show reduced levels of dMyc protein as detected by immunofluorescence (Fig. 3A,A′). Within the wing disc, dMyc protein is normally detected throughout the dorsal and ventral halves of the pouch, with a region of cells along the dorsoventral boundary that express less dMyc^36^. In *en* > *ctp-IR* discs, dMyc staining is intact in the corresponding anterior regions but decreased in these areas that express the *ctp-IR* transgene (Figure S4). Consistent with this link between Ctp and dMyc protein levels, heterozygosity for the null allele *dMyc*^*PL35*^ further shrinks *en* > *ctp-IR* wings (Fig. 3B). In sum these data suggest that reduced cell size contributes significantly to the undergrowth of *en* > *ctp-IR* adult wings, and potentially link *ctp* to one or more of the transcriptional, translational and post-translational mechanisms that control dMyc levels in disc cells^28^,^37^,^38^.

### Ctp is dispensable for multiple signaling pathways but genetically interacts with Yorkie

**Figure 4:**
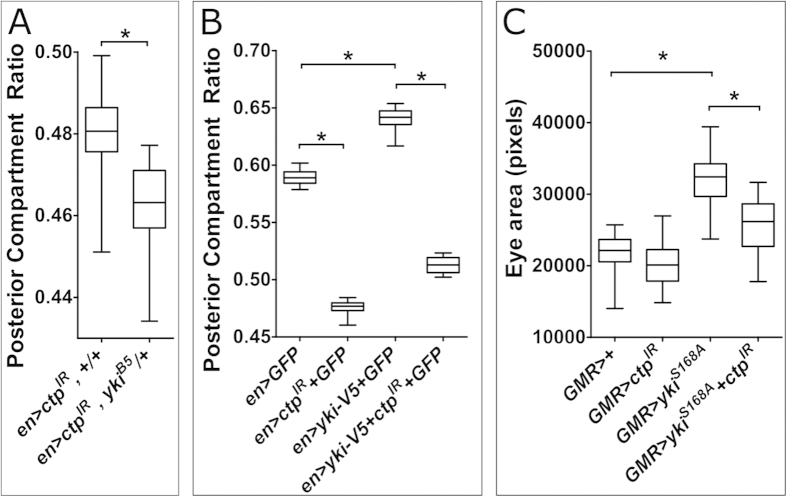
Genetic interactions between *ctp* and *yki* in control of wing and eye size. Box-plot representation of the effect of (**A**) the *yki*^*B5*^ allele (*n* = 13), *p < *0.0001* or (**B**) a *UAS-yki-V5* transgene (*n* = 22, with GFP *n* = 12, with *ctp*^*IR*^ + *GFP*), on PCR in control (*en* > *GFP*, *n* = 12) or Ctp-depleted (*en* > *ctp*^*IR*^ + *GFP*, *n* = 18) adult wings. All *p values < *0.0001*. (**C**) Box-plot showing the effect of *GMR-Gal4* driven Ctp-depletion at 29 °C among eye cells on final adult eye area (2-dimensional *en face* circumference) and the ability of the *ctp*^*IR*^ transgene to suppress this metric in the background of the *GMR-yki*^*S168A*^ hyperactive allele. SEMs are indicated; (GMR, *n* *=* 24; GMR > *ctp*^*IR*^, *n* *=* 22; GMR > *yki*^*S168A*^, *n* *=* 41; GMR > *yki*^*S168A*^ + *ctp*^*IR*^, *n* *=* 24), both *p values < *0.0001*.

Developing imaginal discs are exposed to signals from an array of conserved developmental signaling pathways, some of which are proposed to affect dMyc levels or activity (e.g. Notch and Wg)^39^. Hence, a panel of reagents that detect activity changes in a variety of major cell pathways known to be active in the larval wing were assayed in *en* > *ctp-IR* discs (Figs 3 and S5). These included *Su(H)-lacZ* and *E(spl)-mβ-CD2* (Notch pathway)^40^,^41^, *PCNA-GFP* and *E2f1-lacZ* (E2F/Rb pathway)^42^,^43^, *EcRE-lacZ* (EcR pathway)^44^, *Stat-10xGFP* (Jak-Stat pathway)^45^, and antibodies to the intracellular domain of Notch (N-icd), phospho-Mad (pMad), Wingless (Wg), the Wg/Notch regulated transcription factor Cut^46^, the EGFR pathway component diphospho-Erk (dpErk), and the G1/S regulator CyclinE^47^. None of these markers were evidently altered in the posterior domain of *en* > *ctp-IR* discs (Fig. 3C–K,M) or *ctp*^*ex3*^ clones in the eye disc (Fig. 3L,N). In parallel genetic experiments, a loss-of-function allele of the pro-growth gene *yorkie* (*yki*) was found to dominantly enhance the *en* > *ctp-IR* small-wing phenotype (Fig. 4A). The Yki protein is the main target of the Hippo pathway and acts as nuclear co-activator for Scalloped (Sd)-dependent induction of Hippo target genes, which include *dMyc*^48^. An enlarged-wing phenotype produced by expression of a *UAS-yki-V5* transgene from the *en* > *Gal4* driver is significantly suppressed by co-depletion of Ctp (Fig. 4B). Likewise *ctp* knockdown can suppress eye overgrowth induced by *GMR-Gal4* driven expression of Yki^S168A^, a hyperactive phospho-mutant form of Yki (Fig. 4C). Together, this evidence points towards a functional interaction between *ctp* and *yki* in disc cells destined to form the wing blade and eye.

### Ctp loss reduces *thread/diap1* transcription

**Figure 5:**
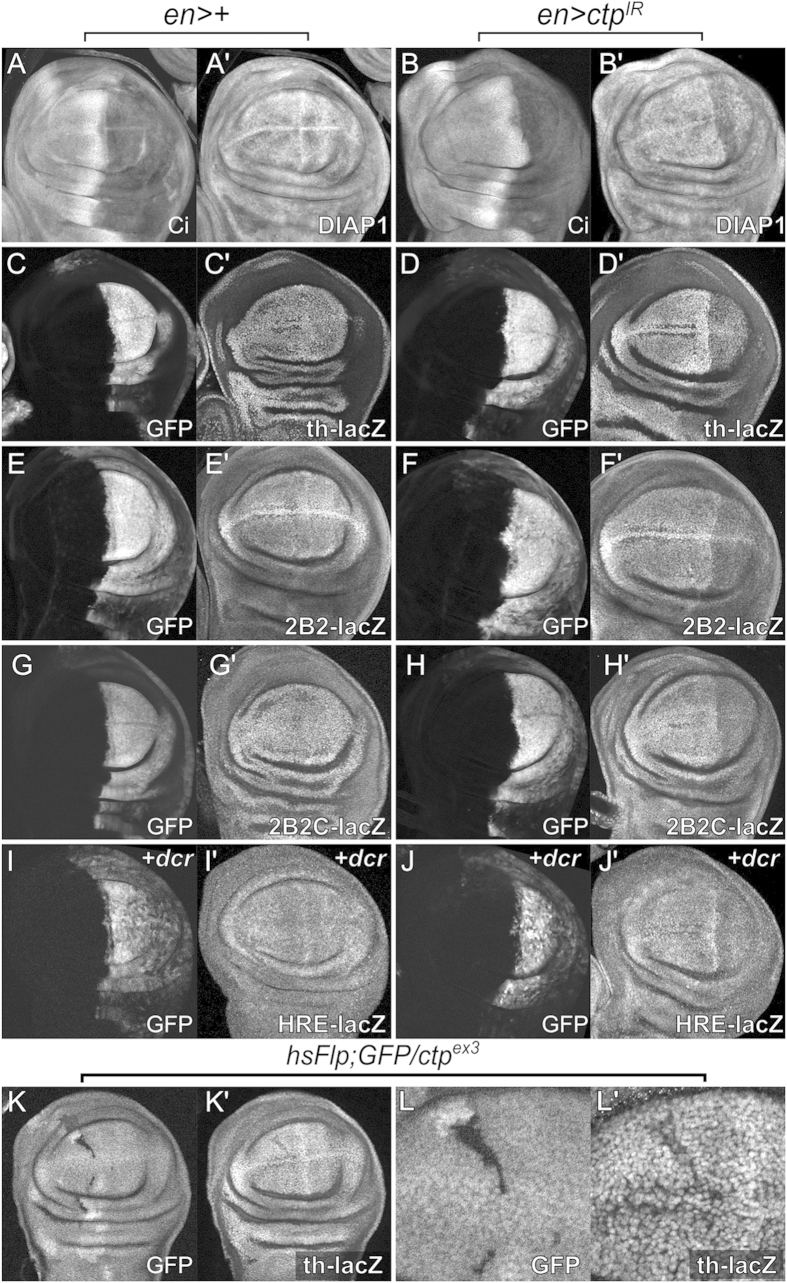
Ctp supports expression of the *diap-1/thread* locus. Confocal images of control (*en* > + in (**A,C,E,G,I**)) or Ctp-depleted (*en* > *ctp*^*IR*^ in (**B,D,F,H,J**)) L3 wing discs stained for Ci (anterior domain) (**A,B**) and Diap1 proteins (**A’,B’**), or with anti-ßgal to detect expression of endogenous *th-lacZ* (**C’,D’**) as well as sequentially smaller Yki-responsive promoter elements *2B2-lacZ* (**E’,F’**), *2B2C-lacZ* (**G’,H’**), and *Hippo response element (HRE)-lacZ* (**I’,J’**). Co-expressed *UAS-GFP* transgene marks the posterior compartment (**C–J**), and the addition of *dicer2 (*+*dcr)* was used to enhance *ctp* knockdown in (**I,J**). Note decreased expression of all three Diap1 markers in cells depleted of Ctp in the posterior (right-side) compartment. (**K,L**) Heat-shock induced GFP-negative *ctp*^*ex3*^ null clones in an L3 wing disc in the background of the *th-lacZ* reporter. Area in L is a magnified view of the anterior clone in K. Note the drop in *th-lacZ* expression in *ctp*^*ex3*^ cells.

The proposed dynein-independent role of mammalian LC8/Ctp as a dimerization hub for cytoplasmic and nuclear complexes (reviewed in refs. 3,7) suggests that Ctp could be involved in modulating activity of the Hippo pathway *in vivo*. This hypothesis was tested by assessing expression of the *thread(th)/diap1* gene, a key anti-apoptosis factor and canonical Yki transcriptional target^23^,^49^,^50^ in Ctp-depleted wing pouch cells. The steady-state level of Diap1 protein is reduced but not eliminated in the posterior compartment of *en* > *ctp-IR* larval discs (Fig. 5A,B), which could explain the mild increase in cC3 signal observed in *ctp* knockdown discs (see Fig. 2A,A′). The *th-lacZ* reporter, which is a Yki-responsive ‘trap’ of the bacterial *β-galactosidase* gene inserted into the endogenous *th/diap1* locus^51^, also shows reduced expression in Ctp-depleted and *ctp*^*ex3*^ mutant cells in the wing pouch (Fig. 5C,D,K,L and Figure S6E,F). A series of successively smaller promoter fragments of the *th/diap1* promoter driving *lacZ* have been used to define a minimal *Hippo response element* (*HRE*) that responds to Yki hyperactivation in larval disc cells^25^. Expression of two of these reporters, *2b2-lacZ* and *2b2c-lacZ*, is strongly reduced in response to *ctp* knockdown (Fig. 5E–H). Baseline expression of the minimal *HRE-lacZ* is fairly low, but its expression is also reduced in Ctp-depleted cells (Fig. 5I,J with co-expression of *dicer2* to enhance *ctp* knockdown, and Figure S6A,B, without *dicer2*). Furthermore, a mutant version of the minimal *HRE* lacking the Sd-binding site necessary for Yki dependent transcription (*HREmut6-lacZ*)^25^ has lowered expression and no longer responds to loss of *ctp* (Figure S6C,D). In sum, these data provide evidence that Ctp is required to support transcription of the Yki target gene *thread/diap1* in larval wing pouch cells.

### Ctp loss elevates transcription of the *bantam* microRNA locus

**Figure 6:**
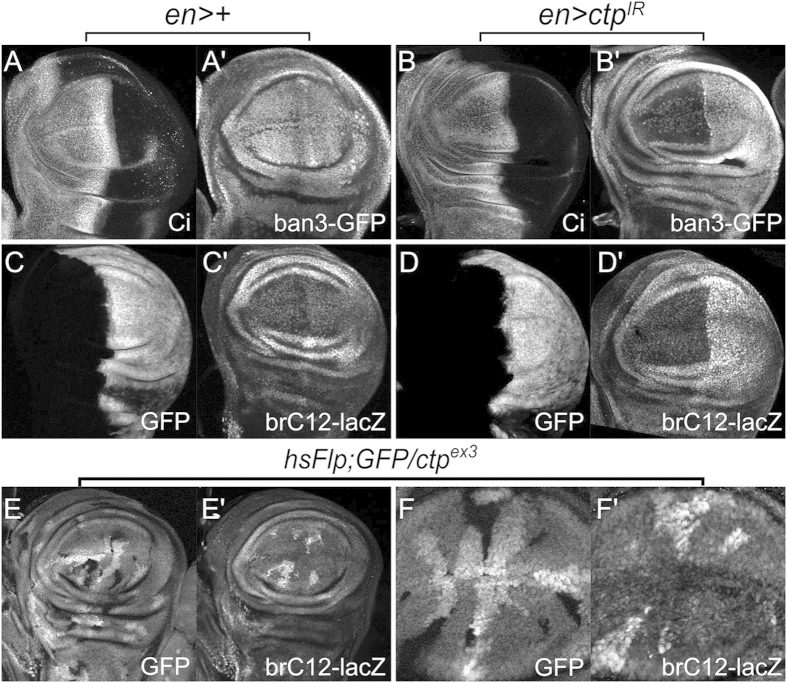
Ctp represses expression of the *bantam* locus. Confocal images of control (*en* > + in (**A,C**)) or Ctp-depleted (*en* > *ctp*^*IR*^ in (**B,D**)) L3 wing discs in the background of the *bantam* reporters *ban3-GFP* (**A’,B’**) or *brC12-lacZ* (**C’,D’**). Anti-Ci marks anterior cells in (**A,B**) while a co-expressed *UAS-GFP* transgene marks posterior cells in (**C,D**). Note increased expression of both *ban3* and *brC12* upon depletion of Ctp in the posterior (right-side) compartment. (**E,F**) Heat-shock induced GFP-negative *ctp*^*ex3*^ null clones in an L3 wing disc in the background of the *brC12-lacZ* reporter. Two different discs are shown. Note consistently elevated *brC12-lacZ* expression in *ctp*^*ex3*^ cells, particularly within the pouch.

Analysis of a second well-validated Yki target, the pro-growth *bantam* (*ban*) microRNA, was carried out to determine whether the requirement for Ctp is unique to *th/diap1* transcription, or can be extended to other well-validated Yki-target genes as well. Two *ban* transcriptional reporter transgenes were used for these studies: *ban3-GFP*, which contains a large proximal fragment of the *ban* promoter driving GFP, and *brC12-lacZ*, a Yki-responsive 410-bp promoter fragment that lies within the *ban3* region and contains two Yki-association sites^27^,^52^. Surprisingly, and in polar contrast to *th/diap1*, expression of both *ban* reporters was strongly elevated in Ctp-depleted wing pouch cells (Fig. 6A–D). The *brC12* reporter is also strongly induced in *ctp*^*ex3*^ null wing pouch clones (Fig. 6E,F; two independent discs are shown with multiple *ctp*^*ex3*^ clones), confirming the link between endogenous Ctp and *ban* promoter activity. Thus, while Ctp normally supports expression of the Yki-response gene *th/diap1* in wing pouch cells, it has the inverse role of repressing transcription of the Yki-responsive locus *ban*. A third classic Yki reporter, *expanded-lacZ*, did not respond to Ctp-loss in otherwise wildtype larval wing cells or in those overexpressing Yki (Figure S7).

### Epistatic relationships between *yki* and *ctp* in control of *th/diap1* and *ban* reporters

**Figure 7:**
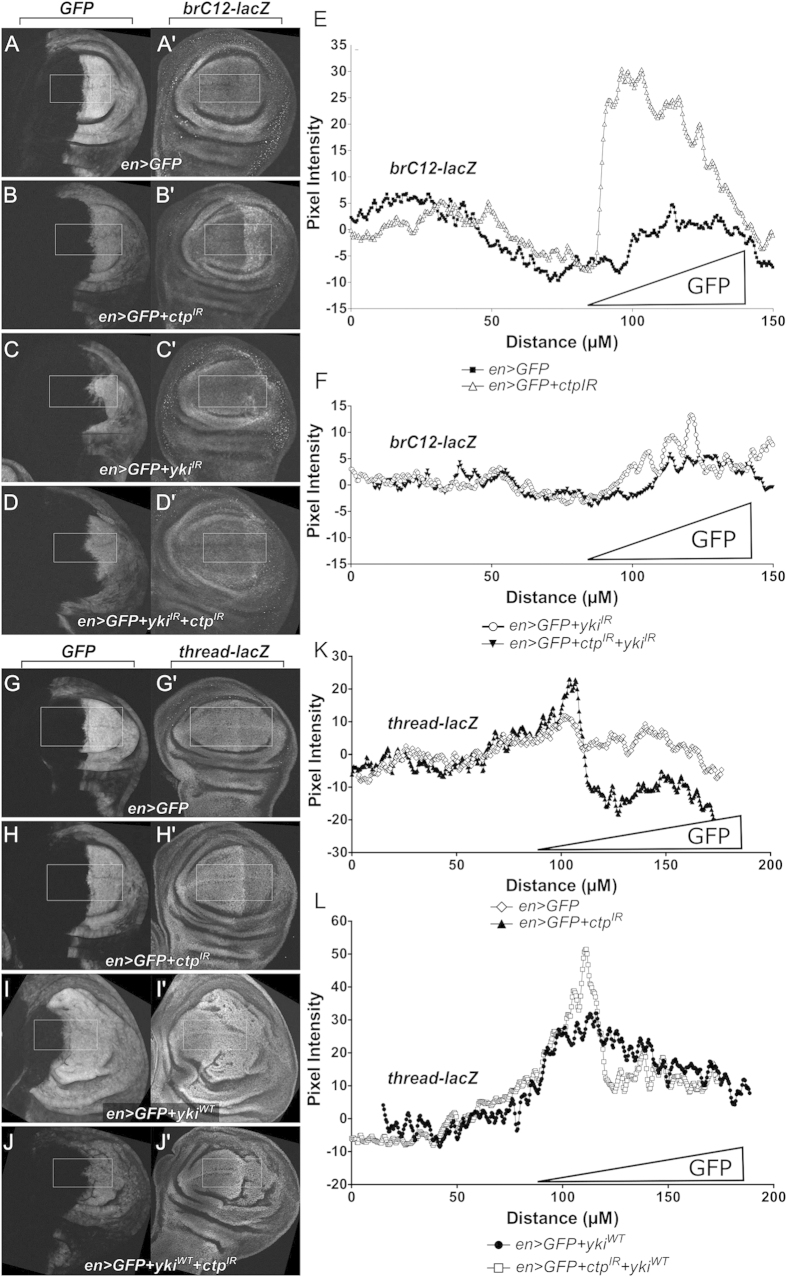
Epistatic relationship between Yki and Ctp-regulated growth. (**A–D**) Anti-βgal staining of L3 wing discs to detect *brC12-lacZ* expression in the background of (**A’**) control *en* > *GFP*; (**B’**) Ctp-depleted, *en* > *GFP* + *ctp*^*IR*^; (**C’**) Yki-depleted, *en* > *GFP* + *yki*^*IR*^; or (**D’**) simultaneous depletion of Yki and Ctp, *en* > *GFP* + *ctp*^*IR*^ + *yki*^*IR*^. Co-expressed *UAS-GFP* transgene marks the posterior compartment (**A–D**). (**E,F**) Quantitation of the effect of Ctp and/or Yki depletion on *brC12-lacZ* expression determined by measuring anti-βgal pixel fluorescence intensity across the anterior:posterior (A:P) boundary (left-to-right) in the white boxes in (**A–D**). GFP-expression domain is indicated. Average fluorescence of the anterior domain was set to zero (0). (**E**) ■ *en* > *GFP*, Δ *en* > *GFP* + *ctp*^*IR*^. (**F**) ○ *en* > *GFP* + *yki*^*IR*^, ▼ *en* > *GFP* + yki^IR^ + *ctp*^*IR*^. (**G–J**) Anti-ßgal staining of L3 wing discs to detect *th-lacZ* expression in the background of (**G’**) control *en* > *GFP*; (**H’**) *en* > *GFP* + *ctp*^*IR*^ Ctp-depleted; (**I’**) *en* > *GFP* + *yki*^*WT*^ Yki-overexpression; or (**J’**) simultaneous expression of Yki and depletion of Ctp, *en* > *GFP* + *yki*^*WT*^ + *ctp*^*IR*^. Co-expressed *UAS-GFP* transgene marks the posterior compartment (**G–J**). (**K,L**) Quantitation of the effect of Ctp depletion and/or Yki overexpression on *th-lacZ* expression determined by measuring anti-ßgal pixel fluorescence intensity across the anterior:posterior (A:P) boundary (left-to-right) in the white boxes in (**G–J**) ⋄ *en* > *GFP*, ▲ *en* > *GFP* + *ctp*^*IR*^. (L) ● *en* > *GFP* + *yki*^*WT*^, □ *en* > *GFP* + *yki*^*WT*^ + *ctp*^*IR*^.

The opposing effects of Ctp loss on the minimally Hippo-responsive *th/diap1* and *ban* promoter fragments imply an Yki-dependent mechanism links Ctp to expression of these genes. To test these relationships, the effect of Ctp loss on *brC12-lacZ* and *th-lacZ* were reassessed either in the presence of overexpressed Yki, or RNAi depletion of endogenous Yki. Elevated *brC12-lacZ* expression upon Ctp-depletion is suppressed by concurrent RNAi depletion of Yki, indicating that Yki is required for maximal induction of *ban* following Ctp loss (Fig. 7A). Furthermore, expression of a *UAS-yki* transgene does not obviously elevate induction *brC12-lacZ* above the level observed in *ctp*^*ex3*^ null clones (Figure S8A,A′). Indeed within the center of the L3 wing pouch, *brC12-lacZ* is more highly expressed in *ctp* null cells than in those expressing a *yki* transgene (Figure S8B; compare centrally located *ctp*^*ex3*^ clone vs. surrounding Yki-expressing cells in the posterior domain). Thus, Ctp-depletion robustly activates *brC12-lacZ* expression in pouch cells via a mechanism that requires Yki. Because overexpression of Ctp in wing pouch cells via *UAS-ctp* has no effect on wing growth or Yki readouts (data not shown), the epistatic relationship between *ctp* and *yki* on the *diap-1* promoter is more challenging to assess. However, transgenic expression of *UAS-yki* remains capable of inducing *th-lacZ* expression in wing disc cells depleted of Ctp (Fig. 7I–L), suggesting that *ctp* acts either upstream or parallel to *yki*. Thus, manipulating Yki levels modifies the effects of Ctp depletion on both *th/diap1* and *ban* reporters, which provides some evidence that *ctp* inversely modulates Yki activity toward each of these target genes.

Wing disc cells lacking the Yki-binding protein Myopic also show selective effects on Yki-target genes^53^. Myopic tethers Yki to endosomes for eventual degradation in lysosomes, and its loss causes Yki to accumulate, leading to induction of *ex* and *ban* but not *thread/diap1*^53^. To test whether Ctp is required for Yki cytoplasmic trafficking in a manner similar to Myopic, endogenous Yki and a V5-epitope tagged form of Yki were visualized in Ctp-depleted pouch cells (Figure S9). Yki steady state levels and nuclear:cytoplasmic distribution are unaltered in Ctp-depleted cells, indicating that wholesale changes in Yki protein dynamics and trafficking in *ctp* mutant wing pouch cells are unlikely to drive downstream effects on *th/diap1* and *ban* expression.

## Discussion

Here we define a role for the *Drosophila* protein Ctp, a member of the LC8 protein family, in regulating expression of two Hippo target genes, *th/diap1* and *bantam*, in larval disc cells. Ctp is a member of a highly conserved family (Ctp, Dlc1, and DYNNL1/DYNNL2) of small proteins that were first identified as components of cytoplasmic dynein motors^1^,^5^,^6^ but are now recognized to also act as interaction hubs for many proteins with roles in diverse processes such as autophagy, signal transduction, cell:cell adhesion, and transcription^3^,^7^,^8^. Largely because of this diverse set of potential effector pathways, the role of LC8 proteins in specific cellular and developmental contexts is not particularly well defined. We find that larval wing compartments depleted of Ctp by RNAi give rise to smaller compartments in the adult wing that are populated by smaller cells. Importantly, the Ctp-depleted larval precursors of these adult wing cells express markers of both M and S-phase, and thus do not appear to undergo cell cycle arrest as reported elsewhere. Parallel analysis with a *ctp* genomic allele confirms that Ctp supports clonal growth in the larval wing disc, particularly in the pouch, and that levels of the pro-growth transcription factor dMyc are reduced in *ctp* mutant epithelial cells. Testing candidate growth regulatory pathways active in the wing pouch uncovers a specific role for Ctp in regulating expression of genes regulated by the Hippo pathway target Yorkie (Yki), which is can positively regulate *dMyc* transcription. Activity reporters of two Yki-responsive genes, *thread/diap1* and *bantam*, each respond to *ctp* loss in pouch cells and these effects map to smaller regions of the *thread/diap1* and *bantam* promoters that contain Yki-responsive elements. Reduction of Yki activity enhances the Ctp-small wing phenotype and depletion of Ctp in turn blunts eye and wing growth driven by *yki* transgenes. These data are consistent with Ctp normally regulating Yki activity and Yki-dependent growth in imaginal disc epithelia.

The effects of Ctp on growth and Yki-target gene transcription are complex and intriguing. While Ctp supports Yki activity on the *thread/diap1* promoter, it restricts activity of the *bantam* promoter, and has no effect on a third Yki reporter, *ex-lacZ*. Moreover transgenic expression of *ban* drives tissue overgrowth^54^,^55^ but upregulation of *ban* transcription in *ctp* mutant cells is paradoxically associated with tissue undergrowth. This apparent *ban* paradox may be explained by the finding that *ban* promotes tissue growth through dMyc^36^,^56^, so that Ctp-depleted cells with reduced dMyc levels may be resistant to *ban*-induced growth.

The opposing effects of Ctp on *thread/diap1* and *ban* transcription differ from core Hippo components, which affect these targets in a uniform way. The mechanism through which Ctp achieves its unique effects on *thread/diap1* and *bantam* is not known. Intriguingly human LC8 interacts with the Kibra protein, a conserved element of the Hippo pathway^15^. *Drosophila* Kibra forms a complex with Merlin and Expanded, and together these proteins promote phosphorylation of Warts^57^. However Warts uniformly represses expression of Yki-target genes, making it unlikely that Ctp acts via a Kibra-Warts axis to exert inverse effects on *thread/diap1* and *ban*. The Kibra-LC8 complex interacts with Sorting Nexin-4 (Snx4) to promote dynein-driven traffic of cargo between the early and recycling endosomal compartments^17^. Yki associates with endosomes^53^ and endosomal traffic modulates levels of Yki protein and the transcription of its nuclear targets^53^,^58^. However, Ctp loss does not obviously alter steady state levels of Yki or its distribution in wing disc cells (see Fig. 7), and although vertebrate LC8 (aka DYNLL1) is as a high-confidence interactor of the Yki vertebrate homolog Yap1^59^,^60^, an equivalent association was not detectable in cultured *Drosophila* S2 cells (data not shown).

In addition to cytoplasmic effects, Ctp/LC8 proteins also have nuclear roles in transcriptional control^3^. For example, LC8 interacts with the estrogen receptor (ER), can promotes ER activity, and is found with ER on the promoters of induced genes^14^. A recent study confirmed that ER dimerizes when bound to DNA and can activate target gene transcription either as a monomer or dimer^61^. *Drosophila* Ctp may thus interact with Yki, or alternatively with a nuclear effector in a distinct pathway that converges on Yki, to bias Yki promoter selectivity and /or to modulate formation of higher order transcriptional complexes with promoter-specific roles *in vivo*. Future studies will be required to identify Ctp interacting proteins within the Hippo pathway and to resolve the precise link between Yki and the multifunctional and highly conserved Ctp/LC8 protein.

## Additional Information

**How to cite this article**: Barron, D. A. and Moberg, K. Inverse regulation of two classic Hippo pathway target genes in *Drosophila* by the dimerization hub protein Ctp. *Sci. Rep*. **6**, 22726; doi: 10.1038/srep22726 (2016).

## Supplementary Material

Supplementary Information
