# Supplementary material for: Inverse regulation of two classic Hippo pathway target genes in Drosophila by the dimerization hub protein Ctp
Source: Sci Rep. 2016 Mar 14;6:22726. doi: 10.1038/srep22726 (PMC4789802; doi:10.1038/srep22726)
Supplement: Supplementary Information [file srep22726-s1.pdf]

Inverse regulation of two classic Hippo pathway target genes in *Drosophila* by the dimerization hub protein Ctp

Daniel A. Barron[1,2] and Ken Moberg[3]
Department of Cell Biology,
Graduate Program in Biochemistry, Cell and Developmental Biology[1],
Medical Scientist MD/PhD Training Program[2],
Emory University School of Medicine
Atlanta, GA 30322, USA

[3]correspondence: kmoberg@emory.edu

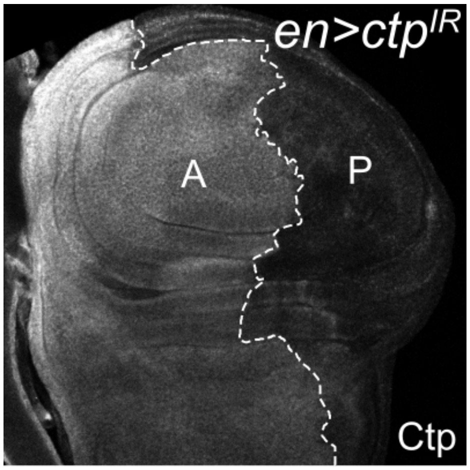

Figure S1. Ctp protein depletion by the *ctp*^{IR} transgene. Confocal image of an L3 *en*>*ctp*^{IR} wing disc immunostained with antiserum to *Chlamydomonas reinhardtii* LC3. Dotted line separates control anterior (A) cells with physiologic levels of Ctp from posterior (P) cells with reduced Ctp levels due to expression of the *ctp*^{IR} transgene.

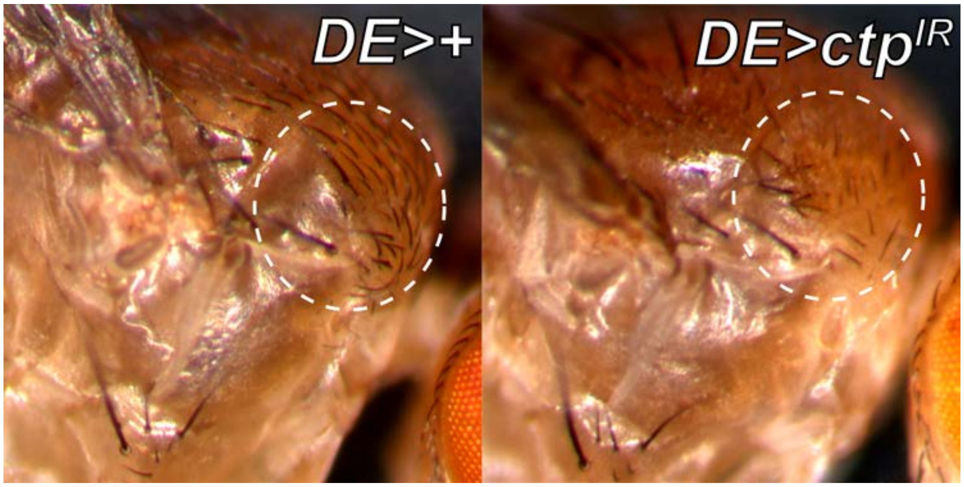

Figure S2. Ctp depletion shrinks the size of thoracic bristles. Light micrograph of humeral bristles (circled) in the callus region of the thorax in control (*DE*>+) or Ctp-depleted (*DE*>*ctp*^{IR}) adult female flies. Note the reduced size of bristles formed from Ctp-depleted cells.

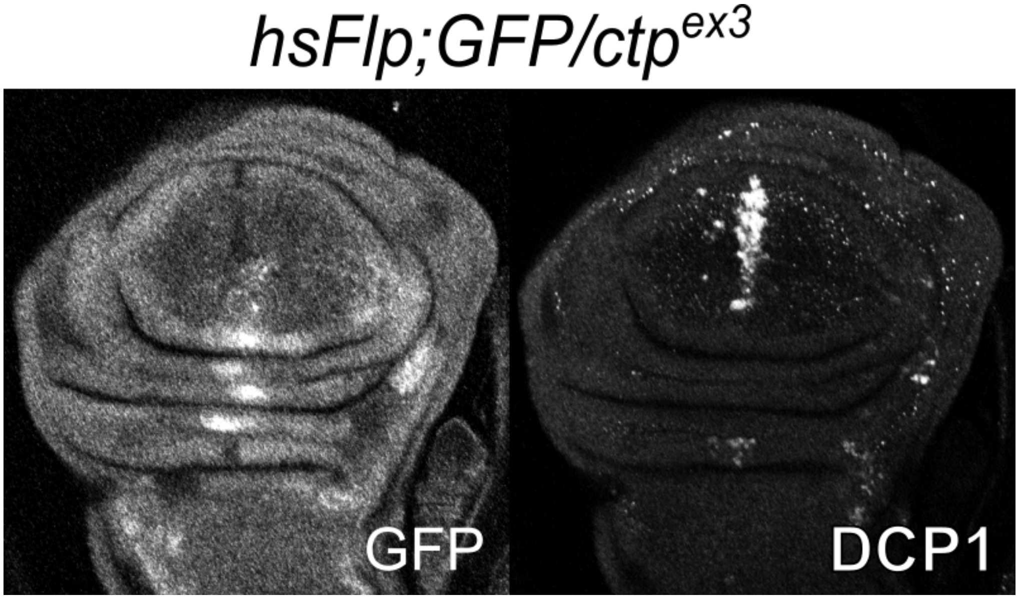

Figure S3. Elevated cleaved DCP1 caspase in *ctp* null wing cells. Confocal image of an L3 wing disc carrying heat-shock induced ctp^{ex3} clones (marked by the absence of GFP) immunostained with an anti-cleaved DCP1 antibody.

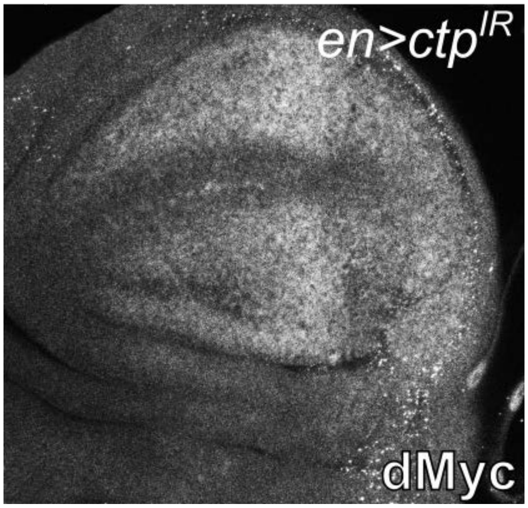

Figure S4. Ctp-depletion lowers level of dMyc in wing cells. Confocal image of an $en>ctp^{IR}$ wing disc stained with an anti-dMyc monoclonal antibody. Note the P-domain (right) is reduced in size and that dMyc protein levels are reduced relative to the control anterior domain.

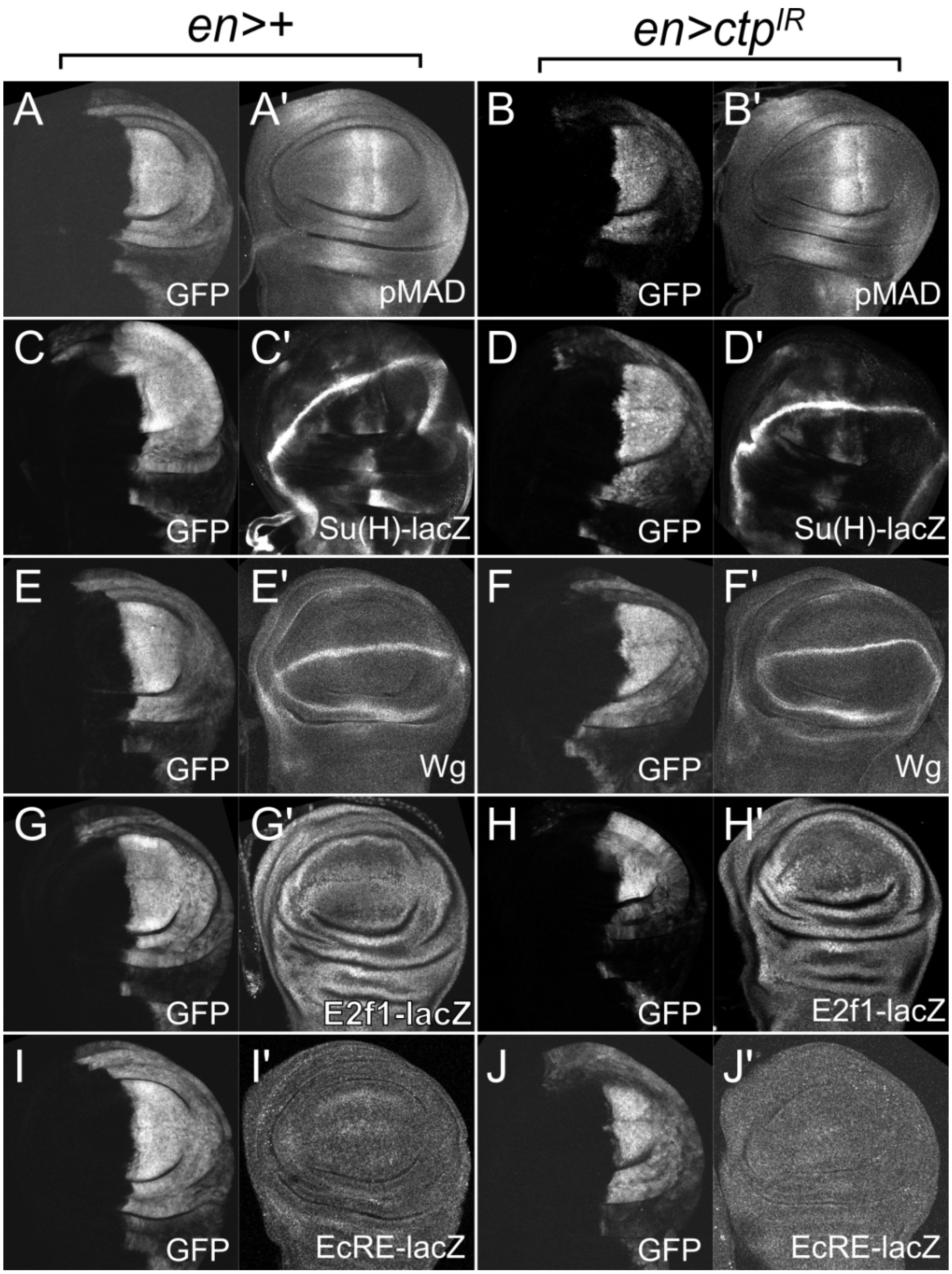

Figure S5. Effect of Ctp loss on a panel of proliferation and/or growth pathways. Paired confocal images of control (A,C,E,G,I) and Ctp-depleted (B,D,F,H,J) L3 wing discs analyzed for each of the indicated factors: (A-B) anti-phospho-MAD (pMAD), (C-D) *Su(H)-lacZ*, (E-F) anti-Wg, (G-H) *E2F1-lacZ*, and (I-J) *EcRE-lacZ*.

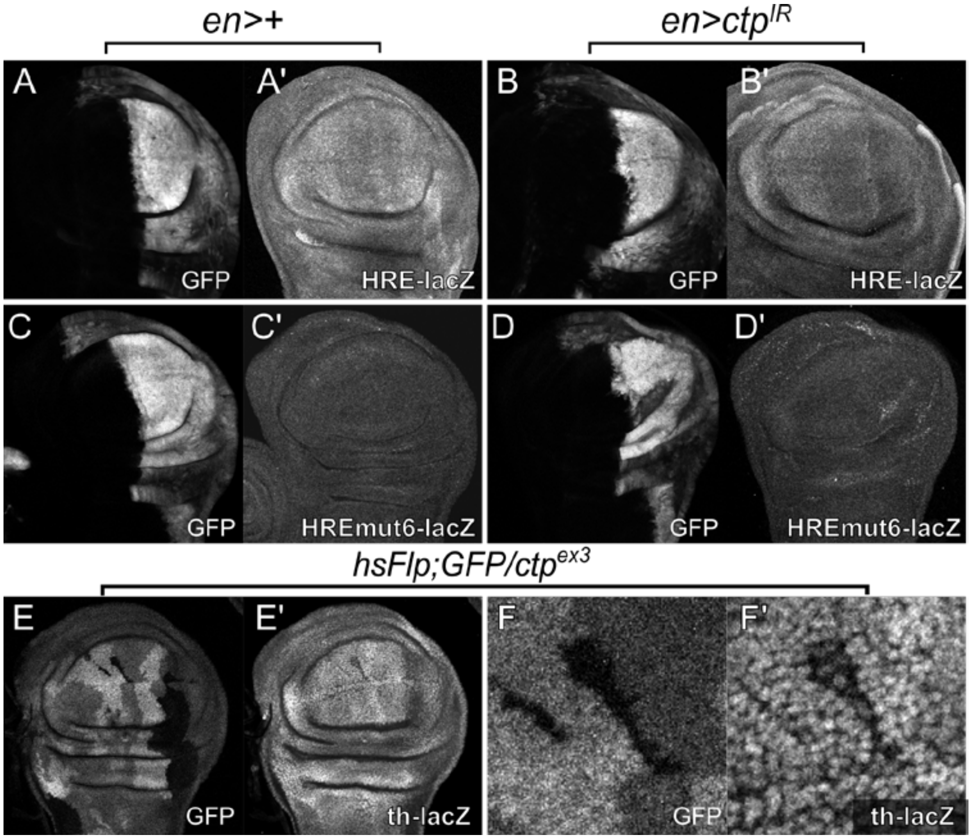

Figure S6. Ctp loss reduces *th* reporter transcription but does not alter Sd-binding site mutant reporter. Paired confocal images of control (*en*>+ in A,C) and Ctp-depleted (*en*>*ctp*^{IR} in B,D) L3 wing discs stained for ßgal to visualize expression of *HRE-lacZ.* (A-B) and *HREmut6-lacZ* (C-D). (E-F) Confocal image of an L3 wing disc carrying heat-shock induced *ctp*^{ex3} clones (marked by the absence of GFP) immunostained with ßgal to detect expression of *th-lacZ.*

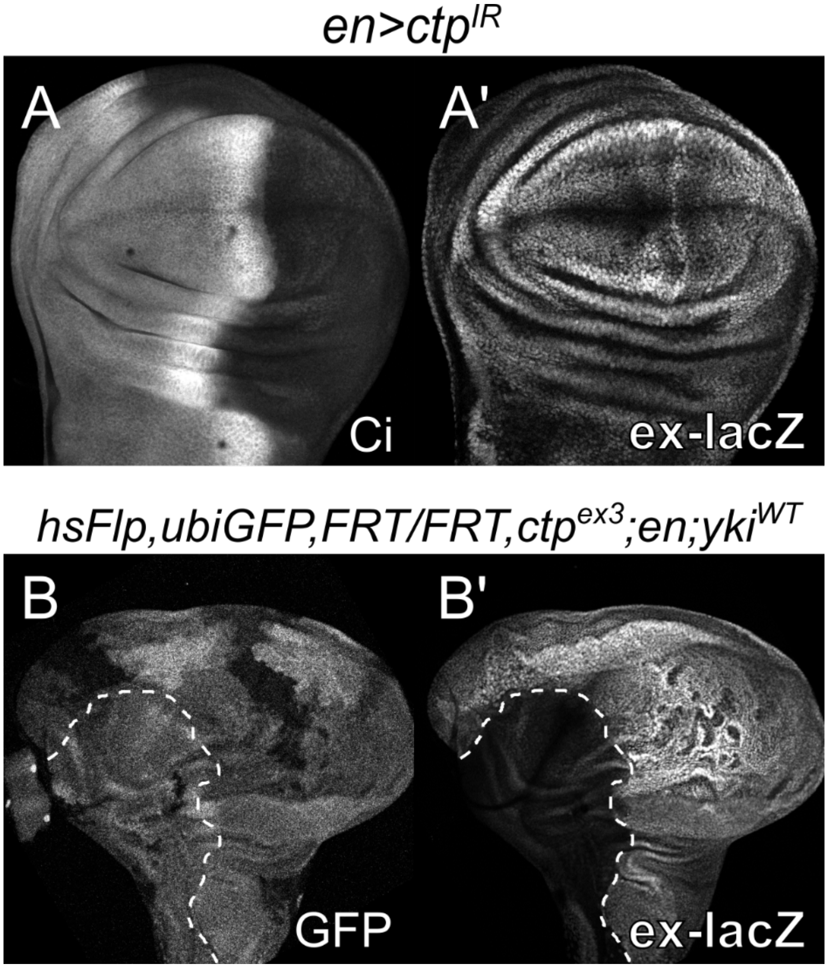

Figure S7. Ctp loss does not affect physiologic or Yki-induced expression of *expanded*. Confocal images of *expanded-lac*Z (*ex-lacZ*) expression in (A-A') a *en>ctp*^{IR} L3 wing disc with Ctp depleted from the Ci-negative posterior domain, or (B-B') a L3 wing disc with GFP-negative *ctp*^{ex3} clones embedded within the control anterior domain (left of dotted line in B) or the Yki-overexpressing posterior domain (right of dotted line in B).

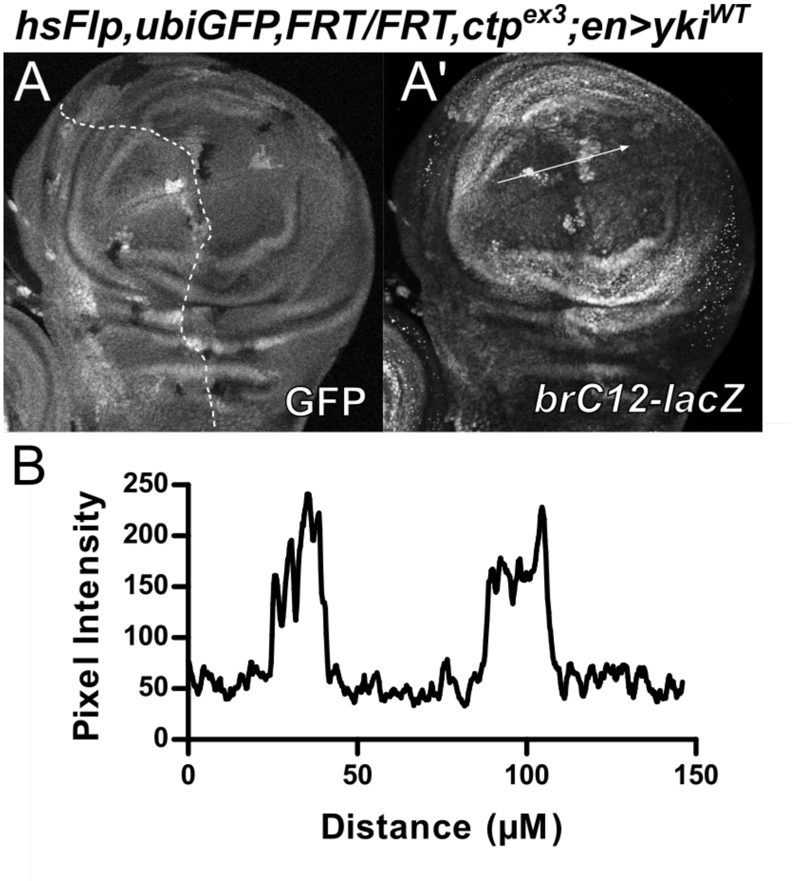

Figure S8. Relative effect of Yki gain and Ctp loss on *brC12* expression. (A-A') Confocal image of an L3 wing disc bearing GFP-negative *ctp*^{ex3} null clones embedded within the control anterior domain (left of dotted line in A) or the Yki-overexpressing posterior domain (right of dotted line in A). The disc has been stained to visualize ßgal expression from the *brC12-lacZ* reporter. Arrow in A' corresponds to the X-axis of the anti-ßgal fluorescence (i.e. pixel) intensity graph in (B), which suggests that *brC12-lacZ* induction in *ctp*^{ex3} clones in both compartments is roughly equivalent.

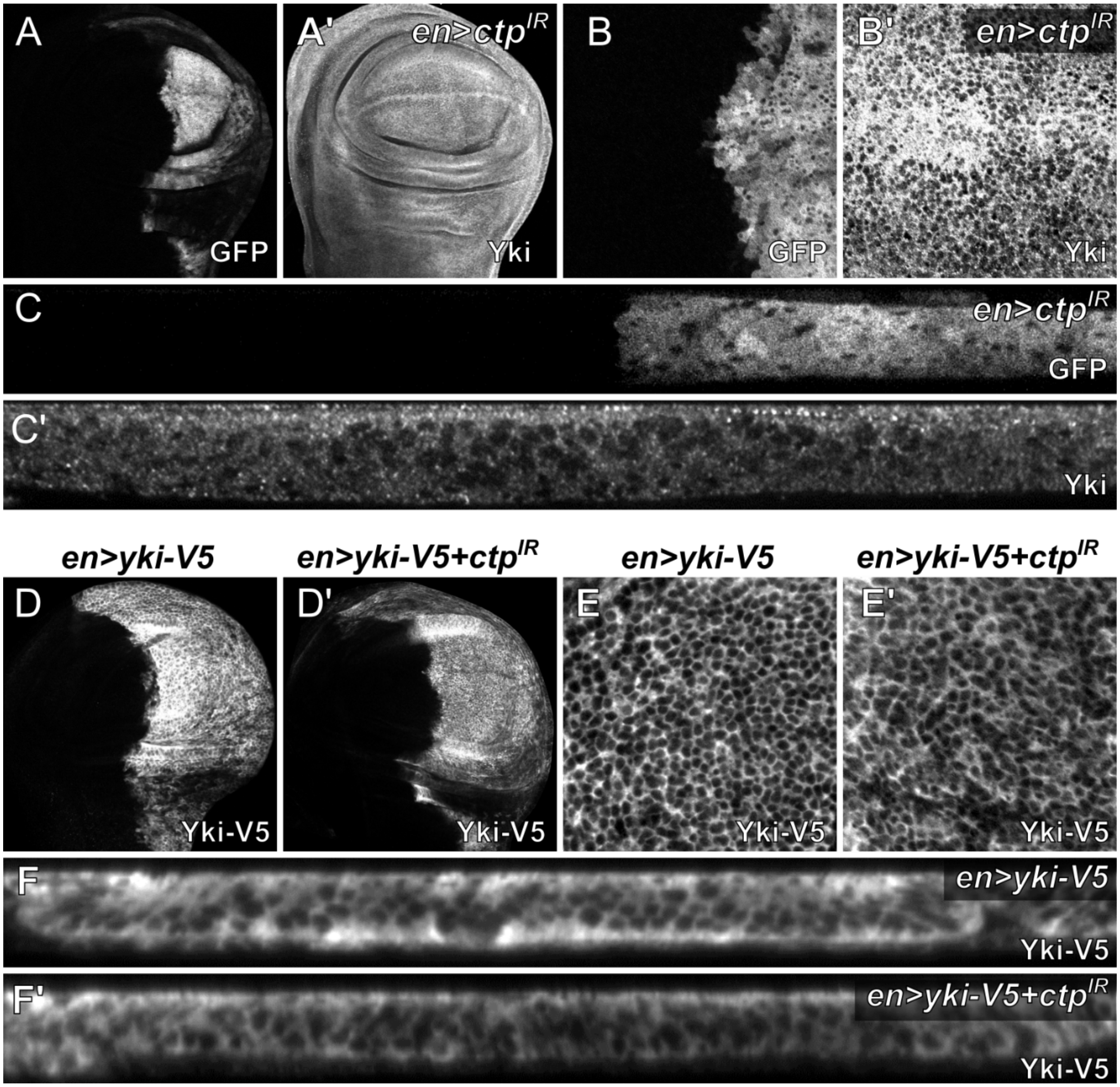

Figure S9. Ctp is not required to control the steady-state distribution or levels of Yki within wing disc cells. Projection images of immunolocalization of (A-C) endogenous Yki or (D-F) transgenic V5-tagged Yki in L3 wing discs lacking Ctp in posterior cells (*en>ctp*^{IR}). GFP and V5 respectively mark the posterior domains in A-C and those in D-F. Magnified and transverse sections along the dorsoventral axis of each pouch are shown in B-C and E-F.
